# Positive Platinum anomalies at three late Holocene high magnitude volcanic events in Western Hemisphere sediments

**DOI:** 10.1038/s41598-018-29741-8

**Published:** 2018-07-26

**Authors:** Kenneth Barnett Tankersley, Nicholas P. Dunning, Lewis A. Owen, Warren D. Huff, Ji Hoon Park, Changjoo Kim, David L. Lentz, Dominique Sparks-Stokes

**Affiliations:** 1 Department of Anthropology and Department of Geology, Braunstein Hall, University of Cincinnati, Cincinnati, Ohio, 45220 USA; 2 Department of Geography, Braunstein Hall, University of Cincinnati, Cincinnati, Ohio, 45220 USA; 3 Department of Geology, Geology and Physics Building, University of Cincinnati, Cincinnati, Ohio, 45220 USA; 4 Department of Geography Education, Kongju National University, Chungcheongnam-do, 32588 South Korea; 5 Department of Biology, Rieveschl Hall, University of Cincinnati, Cincinnati, Ohio, 45221 USA

## Abstract

Changes in the global atmospheric budget of platinum reportedly correspond to explosive volcanic eruptions. Using inductively coupled plasma mass spectrometry (ICP-MS) elemental analysis we examined eight widely separated stratified sites to evaluate the geographic extent of three late Holocene high magnitude volcanic events. We found characteristic Pt anomalies across the Western Hemisphere dating to the Laki, Iceland (CE 1783–1784), Kuwae, Vanuatu (CE 1452–1453), and Eldgjá, Iceland (CE 934) explosive volcanic eruptions. Pt anomalies in sediments over a broad geographic area indicate distinctive time-correlative atmospheric deposition rates of platinum-rich volcanic ash. These anomalies provide new chronostratigraphic markers for these late Holocene high magnitude volcanic eruptions, which are especially valuable in the Western Hemisphere in strata with limited chronometric control. Pt anomalies provide an important tracer for the age of these volcanic events and ultimately a new chronostratigraphic marker in archaeological, geological, palynological, and paleontological sediments.

## Introduction

In 2011, Soyol-Erdene *et al*.^1^ documented atmospheric deposition rates of platinum for the past ~50 years in high summit snow samples collected from two sites in Queen Maud Land, East Antarctica. They sampled snow at 5 cm continuous sequence intervals to a depth of 4 m for Pt concentrations, which were analyzed using inductively coupled plasma mass spectrometry (ICP-MS). Soyol-Erdene *et al*.^1^ discovered an anomalously highly elevated Pt concentration that corresponded to the non-sea salt sulfate (nss-SO_4_) concentration peak of the 1991–1992 Cerro Hudson volcanic eruption. Their finding demonstrates that Pt can be used as a tracer of the aerosol loading of the atmosphere from a high magnitude volcanic event.

Globally, volcanic Pt emission concentrations are significantly higher than in urban air^2^. The magmatic fractionation of Pt is governed by the volatility of Pt-containing complexes (oxides, hydrogen halides, sulfides) and the physicochemical properties of the magma (temperature, fugacities of relevant chemical species). Pt aerosol layers form in the stratosphere after major volcanic eruptions. The dominant Pt aerosol layer is formed by sulfur dioxide gas, which is converted to droplets of sulfuric acid in the stratosphere over the course of a week to several months after the eruption^3,4^. Winds in the stratosphere spread the Pt aerosols until they practically cover the globe and remain in the stratosphere for about two years. Volcanic ash clouds travel along the same pathways as SO_2_ and Pt aerosol particles with a diameter of ∼0.1 mm can be widely distributed by prevailing wind patterns^5^.

Positive Pt anomalies are concentrations greater than the crustal abundance of 0.5 ppb, and these have been used as reliable tracers for internal geological processes such as tectonic movements, faulting, and hydrothermal activity^6,7^. Pt anomalies are also useful as tracers for the accretion of cosmic dust from comets, meteors, and extraterrestrial impacts^6–10^. We investigate sediments from eight late Holocene geomorphic/geologic sites that exhibit no or only minimal signs of bioturbation or other natural or cultural disturbance across the Western Hemisphere where hot springs, faults, and chondrite-rich sediments containing magnetic microspherules and microtektites were absent. These sites allow us to test the occurrence of Pt anomalies at the timing of three high-magnitude late Holocene volcanic events (Supplementary Information). Unlike varved sediments in glacial lakes, undisturbed, well-stratified, and dated geomorphic/geologic sites have a wider geographic distribution and offer more opportunities to examine the occurrence of positive Pt anomalies.

While not all explosive eruptions with a volcanic explosivity index (VEI) ≥5 result in global distributions of tephra, there is causal link between high-magnitude volcanic events and late Holocene climatic change, the most profound of which in terms of human impact is known as the Little Ice Age^11^. Northern latitude tephras tend to remain in the northern hemisphere and tropical latitude eruptions such as Kuwae have global distributions. High-magnitude volcanic activity produces ash and SO_2_, which reaches the stratosphere creating a pan-global ash cloud obstructing solar radiation and results in global cooling. Theoretically, a long-term feedback loop is created when cooled ocean waters and an increase in sea ice result in unusually cold summers^12^. Volcanic ash and SO_2_ from the CE 1452–1453 eruption of the Kuwae volcano in the Republic of Vanuatu and the CE 1783–1784 eruption of the Laki volcano system have been posited as significant contributing factors in the global cooling of the Little Ice Age^11,13^. The ~CE 934 eruption of the Eldgjá volcano in Iceland occurred at the beginning of a warm climatic period in the North Atlantic known as the Medieval Climate Optimum^14^. A climatic warming period may result when significant amounts of volcanic carbon dioxide, a greenhouse gas, are produced.

Kuwae is a submarine volcanic caldera located between the Epi and Tongoa islands. Sometime between late CE 1452 and early CE 1453, Kuwae produced ~32–39 km^3^ of magma and a stratospheric injection of ~175–700 Mt of H_2_SO_4_^14^. Kuwae’s cataclysmic eruption (VEI 7) is considered one of the most explosive volcanic events of the Holocene. Evidence of the Kuwae volcanic eruption is represented in 13 Greenland and 20 Antarctic ice cores as an anomalous sulfate spike^15^. The high magnitude of the Kuwae eruption is based on ~93 kg SO_4_/km^3^ in Antarctica ice cores and ~45 kg SO_4_/km^3^ in the Greenland ice cores^16^.

The Laki volcanic system is located in southern Iceland and includes the Lakagígar volcanic vent or fissure, the Grímsvötn caldera, and the subglacial Thordarhyrna volcano. Explosive eruptions (VEI 6) in the Laki volcanic system occurred between June 1783 and February 1784^16^. During this time, the Laki system produced a convective column of ~120 Mt of SO_2_ into the stratosphere and erupted ~14 km^3^ of basalt lava^16^. The large volume of volcanic ash, water vapor, and reflected solar radiation and absorbed terrestrial radiation resulted in one the longest and coldest drops in historically recorded global temperatures^17^.

Eldgjá is part of the southern Iceland Laki volcanic system, and includes the Katla volcano^18^. Eldgjá’s colossal eruption (VEI 6) originated from a ~200-m deep rift at ~CE 934^18–20^. The eruption produced ~219 Mt of SO_2_, a ~6 km^3^ terrestrial ash fall, and erupted ~19.6 km^3^ of basaltic lava. These are conservative estimations because they do not include ash fall in the ocean or portions of lava flows, which are now covered by late Holocene sediments. Eldgjá was the largest volcanic eruption historically recorded and it produced the largest lava flow during the late Holocene^19^. Written records from Iceland, Western Europe, the Middle East, and Asia document global cooling, famine, and epidemics for ~9 years following the eruption^20^.

We hypothesized that Pt anomalies resulting from three late Holocene high magnitude volcanic events should be present in contemporary sediments across the Western Hemisphere given that Soyol-Erdene *et al*.^1^ found a Pt anomaly in Antarctic snow samples that was associated with a paroxysmic volcanic eruption. Our investigation aims to: a) determine if Pt anomalies from the Laki (CE 1783–1784), Kuwae (CE 1452–1453), and Eldgjá (CE 934) volcanic events might be present in sediments: and b) ascertain if Pt anomalies can be used to distinguish certain high-magnitude volcanic events (VEI ≥ 5) at locations with less precise chronostratigraphic control.

**Figure 1:**
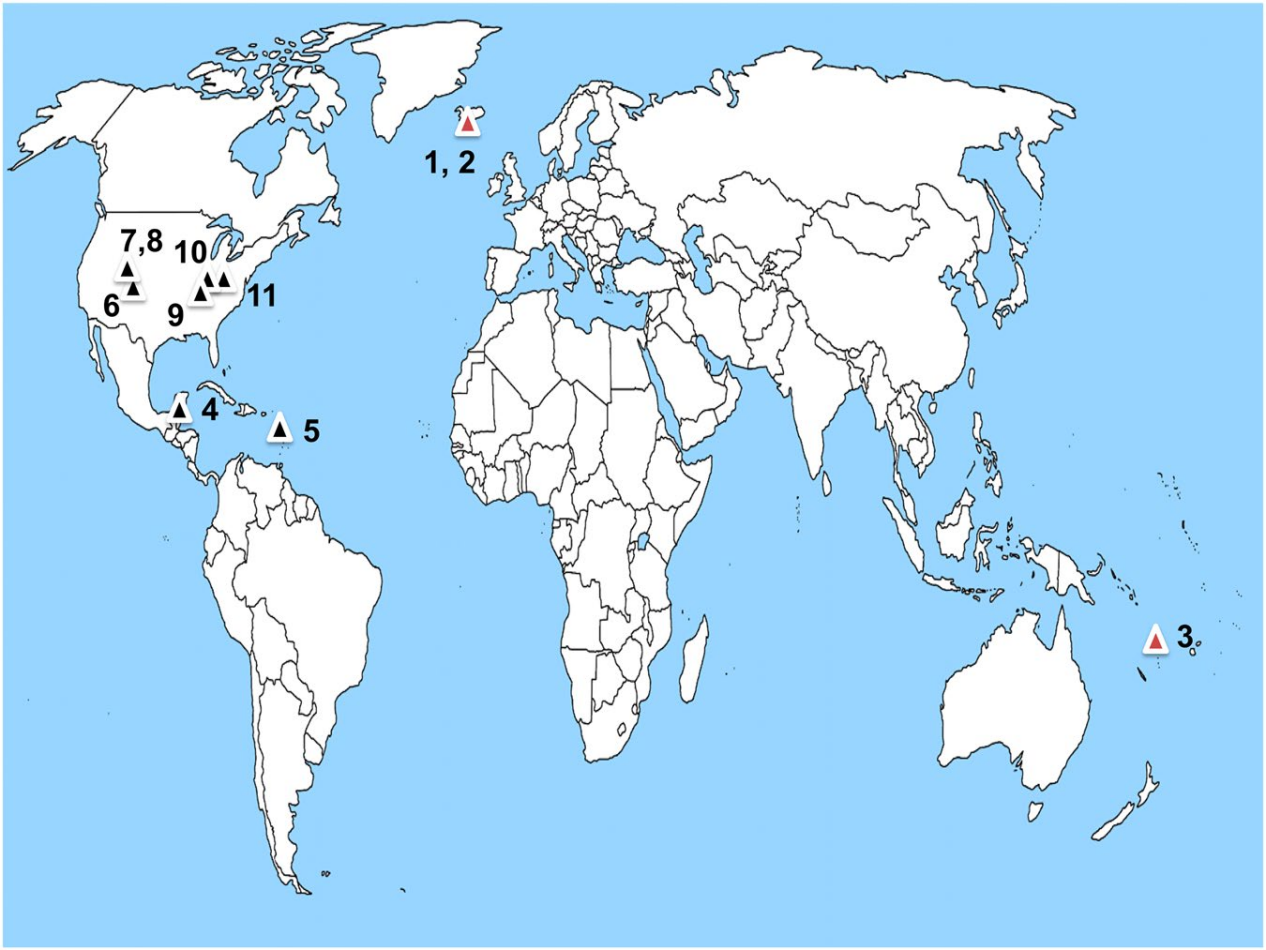
Map showing volcanic centers (red triangles) for late Holocene high-magnitude eruptions (black triangles) for Laki volcanic system (#1), Eldgjá volcanic fissure (#2) and Kuwae volcano (#3). Study sites for platinum (black triangles) include: Temple Reservoir tank, Tikal, Guatemala (#4); Nonsuch Bay, Antigua (#5); Chaco Canyon, New Mexico (#6); Albert Porter Pueblo, Colorado (#7); Wallace Ruin, Colorado (#8); Big Bone Lick, Kentucky (#9); Wynema, Ohio (#10); and Great Serpent Mound, Ohio (#11).

We tested our hypothesis that late Holocene high-magnitude volcanic events would reveal Pt anomalies in sediments obtained from eight well-stratified and chronometrically dated sites across the Western Hemisphere. These sites include: the Temple Reservoir tank at the Maya city of Tikal in the Petén District of northern Guatemala; Nonsuch Bay on the island of Antigua in the West Indies region of the Caribbean; an Ancestral Puebloan canal in Chaco Canyon, New Mexico; the Albert Porter Pueblo and Wallace Ruin, two Ancestral Puebloan Great Houses in southwestern Colorado; Big Bone Lick, Kentucky, a historic contact Fort Ancient bison kill site and a critical geologic site in the historical development of North American Quaternary science and vertebrate paleontology; a sinkhole at Serpent Mound, a 411 m-long earthwork on a karst plateau in southern Ohio; and Wynema, a historic contact Fort Ancient village site in southwestern Ohio (Fig. 1 and Supplementary Information).

We postulated that if these stratified late Holocene sites contained sediments that were deposited at the time of the Laki (CE 1783–1784), Kuwae (CE 1452–1453), and Eldgjá (CE 934) volcanic events, then we should expect to find Pt anomalies. The sediment sample sites have been well-described elsewhere and are also presented in the Supplementary Information. While the sites varied greatly in their age range and geologic setting, all of the sample sites dated to one or more of the late Holocene volcanic events and the sediments were deposited in low energy environments. Sediment samples from the Temple Reservoir tank consisted of aggrading clays^21,22^. The Nonsuch Bay sediment samples consisted of well stratified hemic and sapric organic clays^23^. The American Southwestern samples were poorly consolidated clay, silt, and coarse to medium sand and sandy silty alluvium from Chaco Canyon and a fine-textured silt and fine to medium sandy alluvium overlying an iron-stained clayey silt and fine sandy loess at the Albert Porter Pueblo and Wallace Ruin^24–26^. The Midwestern samples from Big Bone Lick and the Wynema site consisted of a deep and uniformly finely laminated silty alluvium^27^. The Serpent Mound samples consisted of well-stratified silt, silty clay, and clay karst sinkhole deposits^28^.

## Results and Discussion

**Figure 2:**
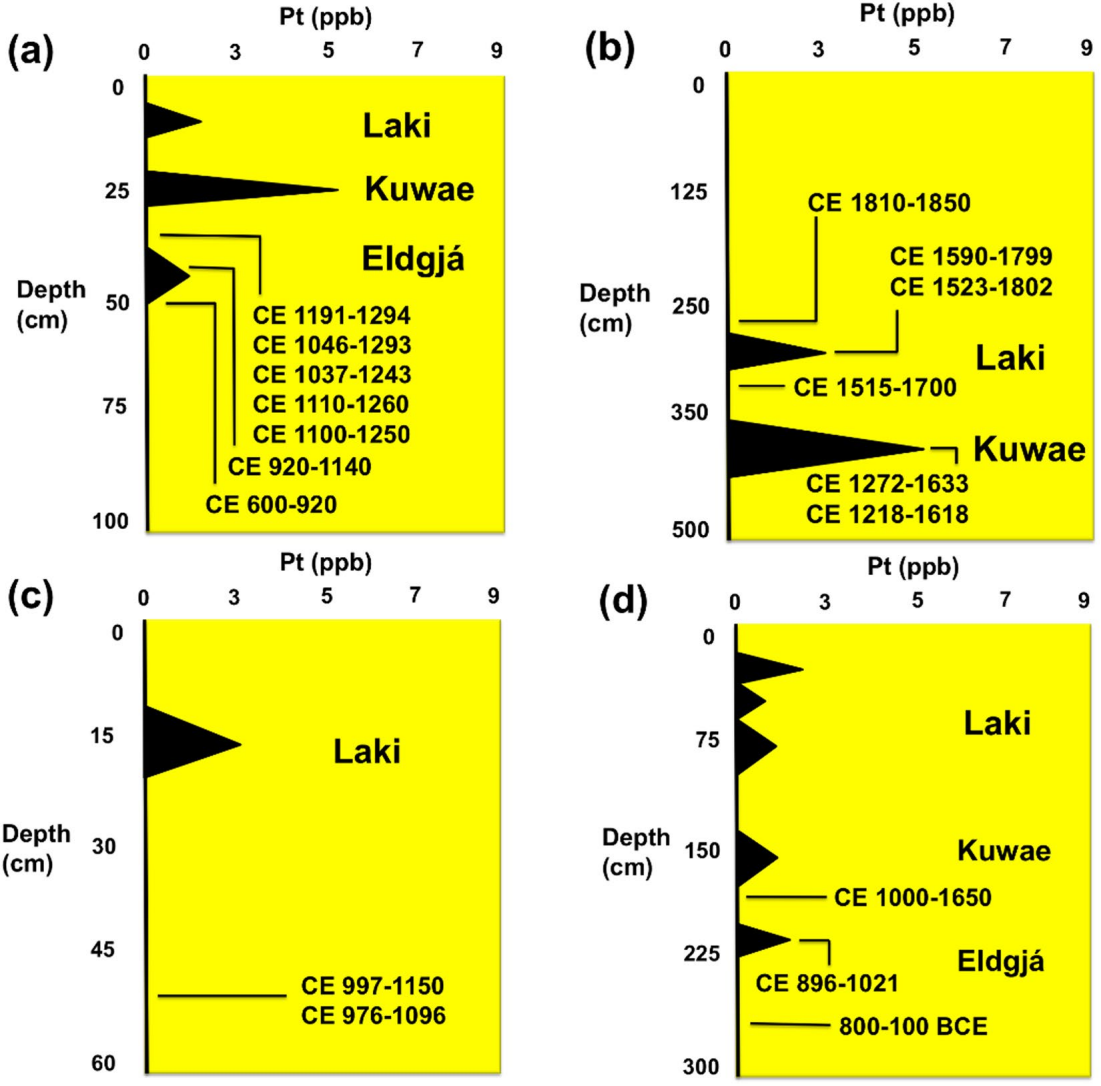
Site graphs for northern latitude (36–39^0^) study sites showing abundance of Pt in ppb (±0.1 ppb), depth, and AMS radiocarbon ages (calibrated years CE with 2 σ uncertainty): (**a**) Albert Porter Pueblo, Colorado; (**b**) Big Bone Lick, Kentucky; (**c**) Chaco Canyon, New Mexico; (**d**) Serpent Mound, Ohio. See Supplementary Information for additional chronostratigraphic details. Zero values represent below detection levels.

**Figure 3:**
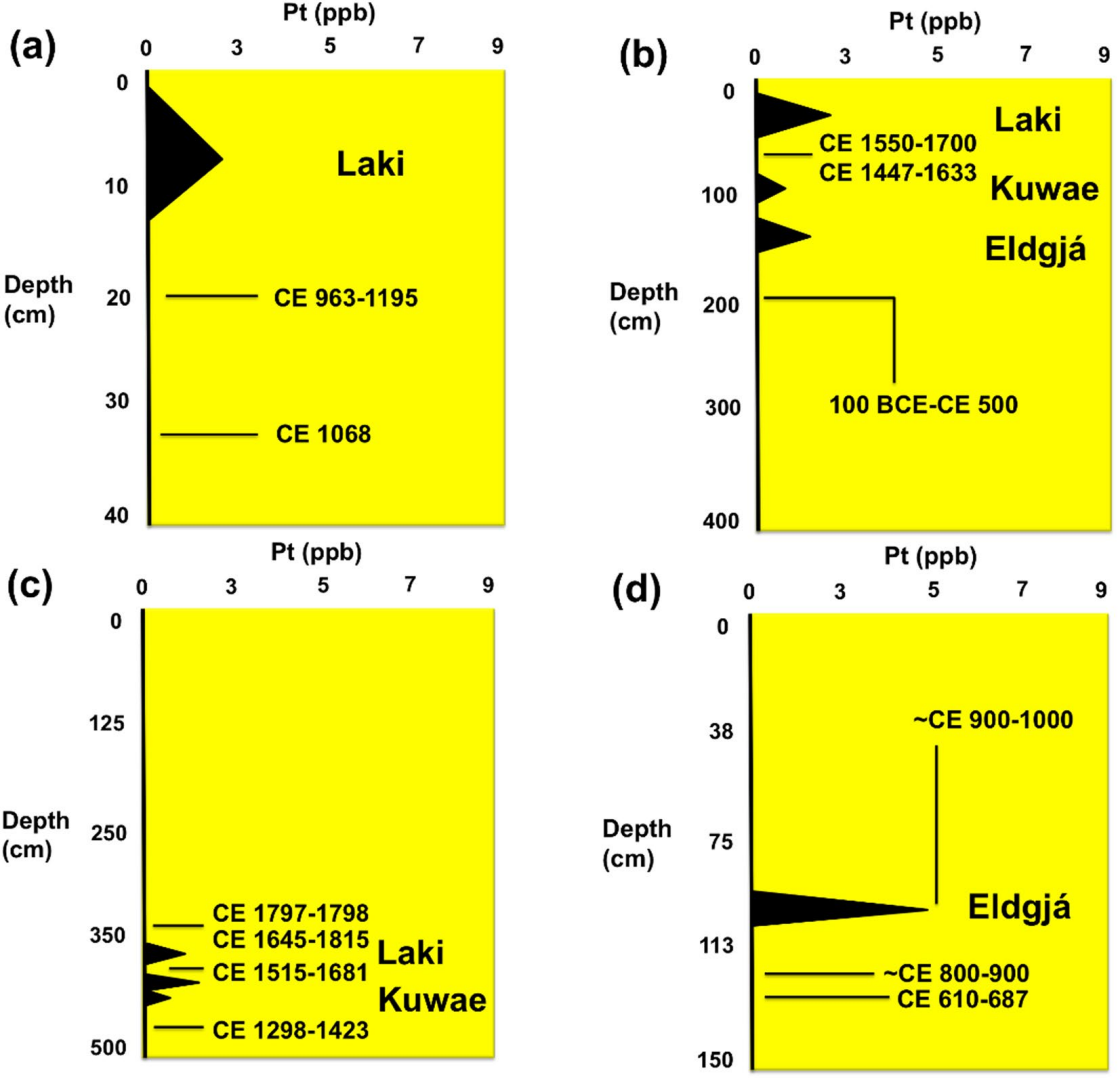
Site graphs for northern latitude (36–39^0^) and tropical latitude (17^0^) study sites showing abundance of Pt in ppb (±0.1 ppb), depth, and radiocarbon ages (calibrated years CE with 2 σ uncertainty); (**a**) Wallace Ruin, Colorado; (**b**) Wynema, Ohio. (**c**) Nonsuch Bay, Antigua, (**d**) Temple Reservoir Tank, Tikal, Guatemala. See Supplementary Information for additional chronostratigraphic details. Zero values represent below detection levels.

The ages of the sediments at our six late Holocene temperate latitude (36–39°N) sample sites in North America (Albert Porter Pueblo, Colorado; Big Bone Lick, Kentucky; Chaco Canyon, New Mexico; Serpent Mound, Ohio, Wallace Ruin, Colorado; Wynema, Ohio; Figs 2 and 3) and our two tropical latitude (17°N) sites (Nonsuch Bay, Antigua; Temple Reservoir tank Tikal, Guatemala; Fig. 3) are based on multiple dating techniques including AMS radiocarbon, optically stimulated luminescence (OSL), dendrochronology, tephra, and artifact typologies. Our analyses identified Pt anomalies at each site in dated strata, which correlated with one or more late Holocene high-magnitude volcanic event.

Our reported results from 39–17^o^ N latitudes and 108–61^o^ W longitudes provide evidence of Pt enrichment in sediments that date to the timing of the high magnitude Laki, Iceland (CE 1783–1784), Kuwae, Vanuatu (CE 1452–1453), and Eldgjá, Iceland (CE 934) volcanic eruptions (Figs 2 and 3; Supplementary Information). Pt anomalies averaged 2.3 ppb at our study sites (range: 1.1 to 5.3 ppb) compared to background abundances (0.0–0.5 ppb) above and below the late Holocene anomalies for these high magnitude volcanic eruptions. They are 5x higher than crustal abundance of 0.5 ppb.

A Pt anomaly was detected in sediments that date to the time of the Laki volcanic system eruption at seven of the sites we sampled (Albert Porter Pueblo, Big Bone Lick, Chaco Canyon, Nonsuch Bay, Serpent Mound, Wallace Ruin, Wynema) and averaged 2.4 ppb and ranged from 1.8 to 2.9 ppb. Another Pt anomaly was found in sediments that date to the time of the Kuwae volcanic eruption at five of the sites we sampled (Albert Porter Pueblo, Big Bone Lick, Nonsuch Bay, Serpent Mound, Wynema) and averaged 2.9 ppb and ranged from 0.6 to 5.2 ppb. A Pt anomaly was also recovered in sediments that date to the time of the Eldgjá volcanic eruption at four of the sites we sampled (Albert Porter Pueblo, Serpent Mound, Temple Reservoir, Wynema) and averaged 2.2 ppb and ranged from 1.1 to 5.1 ppb.

Pt anomalies, which date to the timing of all three of the high-magnitude late Holocene volcanic eruptions, were found in sediment samples from the Albert Porter Pueblo, Serpent Mound, and Wynema sites. Pt anomalies, which correspond to the age of the eruption of the Laki volcanic system, were found in sediment samples from seven of the sites sampled (Albert Porter Pueblo, Big Bone Lick, Chaco Canyon, Nonsuch Bay, Serpent Mound, Wynema). The Pt concentrations between the sites have a relatively small sample variance (0.2), that is, the variation of Pt values. The recent age (CE 1783–1784) of the Laki volcanic event may be the reason for the inter-site consistency of the Pt concentration. That is, it is less likely that post-depositional processes have altered the original Pt content of younger sediments.

Measured concentrations of Pt in late Holocene sediments likely depend upon the distance between the sample site location and the volcano, eruption strength, ash composition, and distribution area of the ejecta. Pt-rich ash, which reached the stratosphere would have had the broadest geographic distribution^1^. Depletion of Pt concentrations at some of the sample sites may have been the result of the size of the site catchment basin, discontinuous deposition, and/or post-depositional erosional processes^7^. Nonetheless, the average Pt anomalies described here for temperate and tropical latitude sites in the Western Hemisphere are relatively consistent in magnitude with regard to the VEI magnitudes of the Laki (mean 2.4 ppb Pt, VEI 6), Kuwae (mean 2.9 ppb Pt, VEI 7), and Eldgjá (mean 2.2 ppb Pt, VEI 6) events. Consequently, Pt concentrations provide an important new tracer for the age of these events and ultimately new chronostratigraphic markers. The widespread distribution of Pt in late Holocene sediments further illustrates the global impact of high magnitude volcanic eruptions, and possibly their role in periods of climatic change such as those experienced during the Little Ice Age.

## Conclusion

Pt anomalies occur in sediments from geographically widely separated sites across the Western Hemisphere, which date to the Laki (CE 1783–1784), Kuwae (CE 1452-1453), and Eldgjá (CE 934) volcanic eruptions. Despite inter-site variances, which likely resulted from post-depositional erosional processes, Pt anomalies provide an effective tracer for certain late Holocene high-magnitude (VEI ≥ 6) volcanic events and ultimately provide three new chronostratigraphic markers on archaeological, geological, palynological, and paleontological sites. The concentrations of Pt from well-dated and well-stratified late Holocene sites provide an opportunity for more vigorous evaluations of the impact of high magnitude volcanic eruptions on climate change and society.

## Methods

Sediment samples were collected from each site in continuous manner by depth. Supplementary Information provides detailed chronostratigraphic information for each of the sites sampled and detailed data are provided for each site related to stratigraphy, age, sampling provenience, and cultural components in Tables 1–17 and Figs 1–3.

Selected aliquots of sediment from late Holocene sites were transferred to pre-weighed digestion vessels. All solutions were prepared with certified trace-metal grade HNO_3_ (67–70% w/w) and HCl (36% w/w) and ultra-pure (18MΩ) water. Sediment aliquots were homogenized and digested with Aqua Regia (3:1 HCl:HNO_3_ mol/mol) in Savillex PFA containers and heated at 90 °C for 1 hour on a heating block. After cooling, the solutions were then diluted with 18 MΩ water and analyzed by ICP-MS^29^. The certified reference material (SARM-7, SACCRM) was digested using the same procedure as a means of corroboration. The value of the certified reference material (SARM-7, SACCRM) was 3.74 ± 0.05 ppm and the measured value was 4.27 ± 0.13 ppm.

ICP-MS analyses were completed on a Thermo Scientific X Series II instrument. A peristaltic pump using a Cetac ASX 520 auto-sampler pumped sample solutions. The internal standard was added in-line using a Trident Internal Standard Kit. The sample was introduced into the plasma using a MicroMist EzyFit nebulizer, which reduced oxide formation with a high total dissolved solids tolerance, and reduced the sample uptake rates. The cyclonic spray chamber was kept at 3 °C, minimizing oxide formation. Ion lens voltages, nebulizer flow, and stage positioning were optimized every 24 hours using a tuning solution to maximize the ion signal and stability and minimize oxide levels (CeO^+^/Ce^+^) and doubly charged ions (Ba^2+^/Ba^+^). A calibration check of the standards was analyzed following initial calibration, at the end of the sample run, and after every 12 samples.

### Data Availability

All data generated or analyzed during this study are included in this published article (and its Supplementary Information files).

## Electronic supplementary material

Supplementary Information
