# Supplementary material for: Positive Platinum anomalies at three late Holocene high magnitude volcanic events in Western Hemisphere sediments
Source: Sci Rep. 2018 Jul 26;8:11298. doi: 10.1038/s41598-018-29741-8 (PMC6062578; doi:10.1038/s41598-018-29741-8)
Supplement: Supplementary file 1 — Supplementary Information [file 41598_2018_29741_MOESM1_ESM.docx]

**Supplementary Information: Site Data, Pt Aerosols, and Tephra Volume**

**Positive Platinum anomalies at three late Holocene high magnitude volcanic events in Western Hemisphere sediments**

Kenneth Barnett Tankersley*^1,2^, Nicholas P. Dunning^3^, Lewis A. Owen^2^, Warren D. Huff^2^,

Ji Hoon Park^4^, Changjoo Kim^3^, David L. Lentz^5^, Dominique Sparks-Stokes^1^.

^*1^Department of Anthropology, University of Cincinnati, Cincinnati, Ohio, 45221, USA. ^2^Department of Geology, University of Cincinnati, Cincinnati, Ohio, 45221, USA.

^3^Department of Geography, University of Cincinnati, Cincinnati, Ohio, 45221, USA. ^4^Department of Geography Education, Kongju National University, Chungcheongnam-do, 32588, Korea.

Table 1. Site Pt peak data summary^a,b^.

| **Sample Site** | **Laki**  **(Pt ppb)** | **Kuwae**  **(Pt ppb)** | **Eldgjá**  **(Pt ppb)** |
| --- | --- | --- | --- |
| Albert Porter Pueblo, Colorado | 1.8 | 5.3 | 1.4 |
| Big Bone Lick, Kentucky | 2.3 | 4.7 | nd |
| Chaco Canyon, New Mexico | 2.8 | nd | nd |
| Nonsuch Bay, Antigua | 2.6 | 2.6 | nd |
| Serpent Mound, Ohio | 1.8 | 1.1 | 1.2 |
| Temple Reservoir Tank, Guatemala | nd | nd | 5.1 |
| Wallace Ruin, Colorado | 2.5 | nd | nd |
| Wynema, Ohio | 2.9 | 0.6 | 1.1 |
| **Range** | 1.8–2.9 | 0.6–5.3 | 1.1–5.1 |
| **Mean** | 2.4 | 2.9 | 2.2 |

a. nd = no data.

b. SARM-7 certified value was 3.74 ± 0.05 ppm and the measured value was 4.27 ± 0.13 ppm.

**Antigua**

*Nonsuch Bay*

Nonsuch Bay is a prominent embayment on the eastern side of the island of Antigua, West Indies (Figure 1). Core NS07-2 was collected as part of a multi-island paleoecology project that examined the pattern of human migration and environmental disturbance in the Lesser Antilles^1,2^. The 456-cm-long core was retrieved with a modified Livingston piston corer in a mangrove-dominated estuary where Ayres Creek discharges into the bay. The stratigraphy of the core exhibited great integrity with only minimal signs of bioturbation or other disturbance (e.g., a small amount of mangrove root penetration between 390 and 405 cm. depth). The portions of the core discussed in this article are illustrated in Figure 1; the entire core is described in detail elsewhere.^2^ A radiocarbon age of 580 + 35 BP (calibrated to 1300–1420 CE; all radiocarbon ages in this supplement are calibrated using CALIB 7.1 and the IntCal13 calibration) was obtained from “sapric clay” (clay with between 5-10% sapric organic matter) sediment from 445 cm (Table 2, Figure 1). Data from the core and from survey in the contributing watershed indicates catastrophic soil erosion associated with Colonial era sugarcane cultivation is responsible for most of the sediment in the estuary^3^. The samples reported here are from between depths of 349 and 439 cm in the core (Table 3). Most of the core in this section consists of clay with a high organic matter content. Lenses of volcanic ash at 351−348 cm and 362−360 cm likely correspond to the CE 1797−1798 eruption of La Grande Soufrière on the island of Guadeloupe which is reported to have resulted in significant ash fall on Antigua. The samples reported here straddle the lower and middle of three pollen zones identified in the NS07-02 core, that is, the transition from the pre-Columbian and earliest Colonial eras to the era of peak Colonial plantation agriculture with its associated deforestation and soil erosion.^2^ These pollen data are consistent with the radiocarbon-based chronology.

Figure 1. A cross-section of the Nonsuch Bay, Antiqua core and site location.


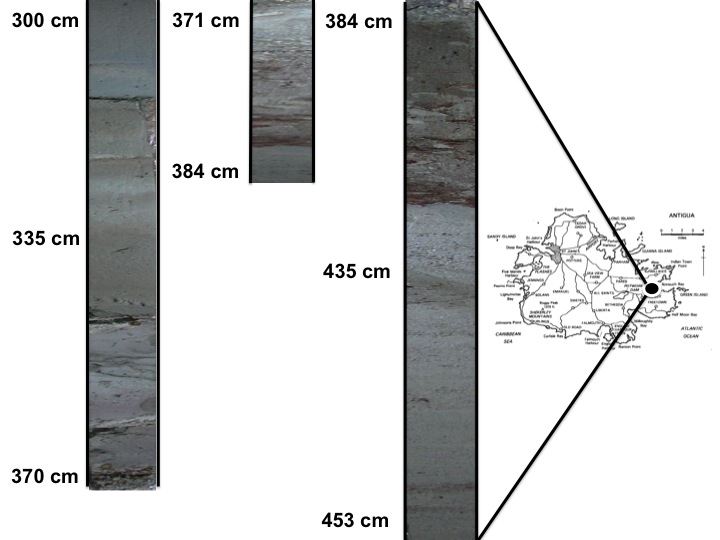


Table 2. Radiocarbon and tephra ages for Nonsuch Bay, Antiqua.

| **Sample** | **Lab #** | **^14^C age yr BP** | **Cal age (2 σ)^a^** | **Probability** |
| --- | --- | --- | --- | --- |
| Tephra |  |  | CE 1797−1798 |  |
| Organic Matter | AA82476 | 191 ± 38 | CE 1645−1697  CE 1724−1815  CE 1834−1878  CE 1916−1950 | 0.240  0.531  0.059  0.169 |
| Organic Matter | AA82475 | 254 ± 36 | CE 1515−1598  CE 1617−1681  CE 1739−1745  CE 1762−1802  CE 1937−1950 | 0.277 0.495  0.005  0.187  0.035 |
| Organic Matter | AA77643 | 577 ± 37 | CE 1298−1371  CE 1379−1423 | 0.641  0.359 |

1. CALIB 7.1 and the IntCal13 Calibration.

Figure 2. Bayesian adjustments of the radiocarbon ages using OXCAL to give the full range of possible ages for Nonsuch Bay, Antiqua.


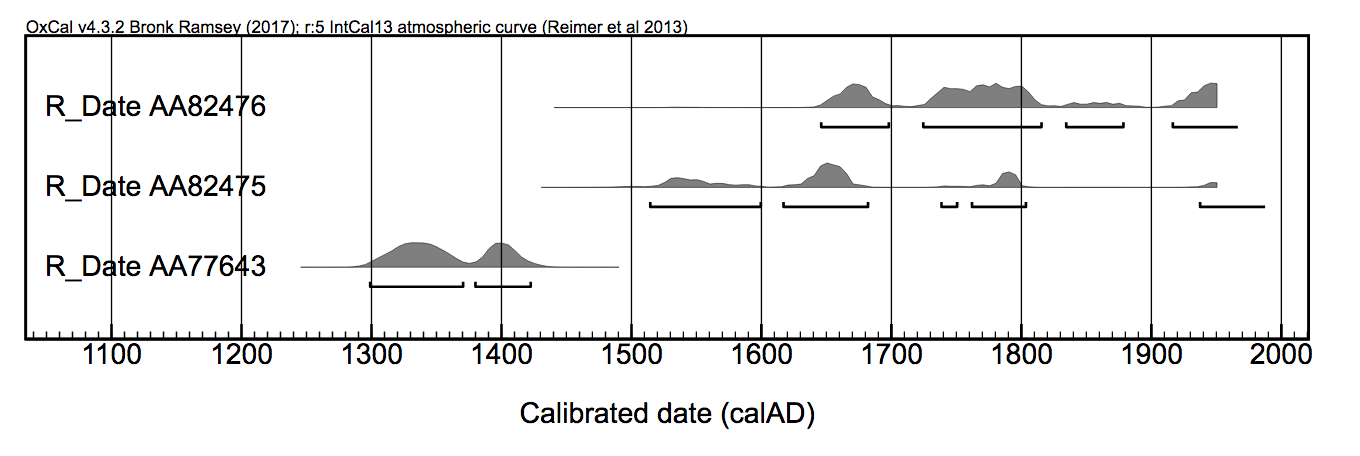


Table 3. Chronostratigraphy and Pt values for Nonsuch Bay, Antiqua.

| **Depth (m)** | **Composition** | **Munsell Soil Color** | **Age** | **Pt (ppb)^a^** |
| --- | --- | --- | --- | --- |
| 3.51-3.60 | Banded Sapric clay | Gley 10Y 5/1 | CE 1797−1798 | nd |
| 3.49 | Sapric clay | Gley 10Y5/1 | CE 1645−1815 | nd |
| 3.90 | Hemic peat | Gley 10Y2.5/1 |  | 2.6 |
| 3.98 | Hemic peat | Gley 10Y2.5/1 | CE 1515−1681 | nd |
| 4.20 | Sapric clay | Gley 10Y3/1 |  | 2.6 |
| 4.39 | Sapric clay | Gley 10Y3/1 |  | 1.8 |
| 4.45 | Sapric clay | Gley 10Y3/1 | CE 1298−1423 | nd |

a. nd = no data

**Guatemala**

*Temple Reservoir Tank, Tikal,*

The Temple Reservoir Tank is located in the heart of an area of monumental architecture in “downtown” Tikal, an ancient Maya city in the Peten District of Guatemala (Figure 3). This feature lies above the much larger Temple Reservoir proper. Originally thought to be a silting tank to protect water quality in the Temple Reservoir, excavations in 2009 revealed that the tank seems to have been designed to collect water from a now plugged natural spring^4,5^. Excavations and coring in the tank indicated that it was periodically dredged, but seems to have steadily accumulated clayey sediment from the Seventh century CE onward (Table 4). Operation 7A was a 1 x 1 m excavation that penetrated to sterile substrate at a depth of about 2.2 m. Strata revealed within the profile showed little disturbance from either bioturbation or argilloturbation (clay heaving), both of which sometimes disturb strata in the Maya Lowlands. The charcoal sample reported here was collected from the pit wall at a depth of 130 cm; the analyzed sediment sample was collected from the pit wall at a depth of 100 cm (Table 5), which should correspond to the period between CE 900 and 1100. Although abundant ceramic sherds were recovered in Op. 7A, almost all were too weathered for chronological identification (typical of reservoir sherds). Between a depth of 125 and 150 cm, sherds were extremely abundant and seem to correspond to the Late Classic period (CE 600-800), consistent with the radiocarbon date. Between a depth of 125 and 100 cm, sherds diminished dramatically in number and those identifiable were a mixed lot of Classic Period types, consistent with Terminal Classic and post-abandonment deposits (i.e., consistent with a 9^th^ century chronological date). Only a handful of unidentifiable sherds were recovered between 85 and 100 cm (consistent with post-abandonment deposits; i.e., a 10^th^ century CE date)^6,7^.

Figure 3. Profile of the Tikal, Guatemala excavation and site location.


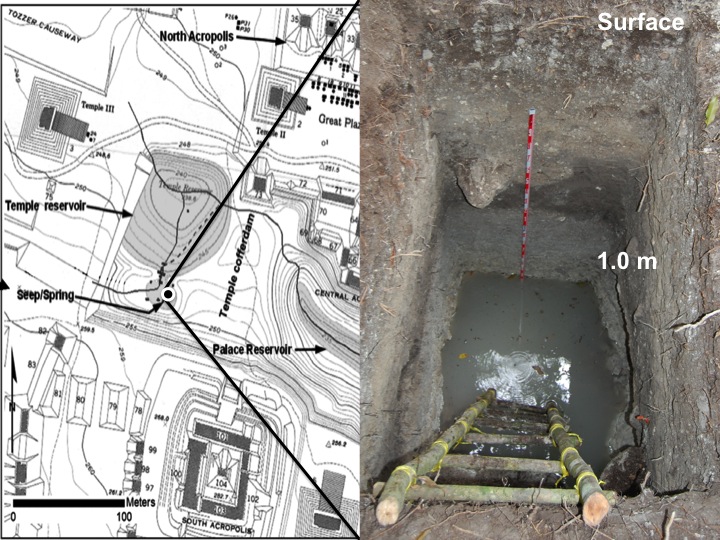


Table 4. Radiocarbon and ceramic typological ages for Temple Reservoir Tank, Tikal.

| **Sample** | **Lab #** | **^14^C age yr BP** | **Cal age (2 σ)^a^** | **Probability** |
| --- | --- | --- | --- | --- |
| Post-abandonment Refuse |  |  | ~CE 900-1000 |  |
| Terminal Classic Mixed Ceramic Deposit |  |  | ~CE 800−900 |  |
| Late Classic ceramics |  |  | ~CE 600-800 |  |
| Wood Charcoal | Beta 298985 | 1370 ± 30 | CE 610−687 | 1.000 |

1. CALIB 7.1 and the IntCal13 Calibration.

Table 5. Chronostratigraphy and Pt values for Temple Reservoir Tank, Tikal.

| **Depth (m)** | **Texture** | **Munsell Soil Color** | **Age** | **Pt (ppb)^a^** |
| --- | --- | --- | --- | --- |
| 1.0 | Silty clay | 7.5YR6/1 | ~CE 900-1000 | 5.1 |
| 1.25 | Silty clay | 7.5YR7/1 | ~CE 800−900 |  |
| 1.3 | Silty clay | 7.5YR7/1 | CE 610−687 | Nd |

1. nd = no data.

**USA**

*Albert Porter Pueblo, Colorado*

Albert Porter Pueblo is an Ancestral Publoan village located on a mesa top between Sandstone and Woods Canyons in southwestern Colorado (Figure 4). The site includes Chacoan masonry, 28 multistory rooms, 26 kivas, 6 pit structures, and 54 midden deposits. Dendrochronology samples obtained from the Albert Porter Pueblo span from ~CE 860−1260 and cutting ages for the wood suggest that the site was occupied continuously from ~ CE 1110−1260^8^. Radiocarbon ages are consistent with the dendrochronology (Table 6, Figure 5). The oldest ceramic artifacts from the Albert Porter Pueblo date to the Basketmaker III and Pueblo I cultural periods (~ CE 600–920). The midden deposits are dominated by ceramic artifacts, which date to the Pueblo II and Pueblo III (~ CE 920–1140) cultural periods with the greatest concentration of pottery dating to ~ CE 1100–1250^8^. Sediment samples were obtained from a solid sediment core extracted from the open plaza of the pueblo. Natural stratigraphy was defined on the basis of soil texture and color (Table 7). Sediment samples were collected from the open-air plaza portion of the site with intact stratigraphy. There was no evidence of bioturbation or other natural or cultural disturbances. The stratigraphy discussed in this article is illustrated in Figure 5 and the entire site is described in detail elsewhere^8^.

Figure 4. Profile of the Albert Porter Pueblo, Colorado excavation and site location.


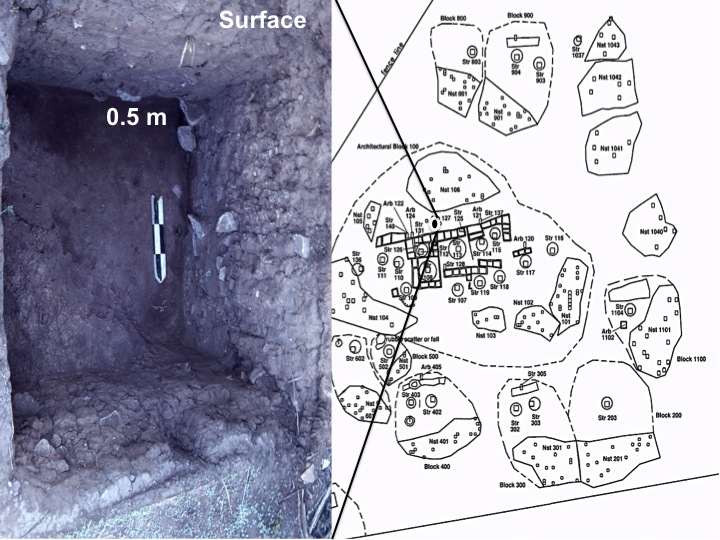


Table 6. Radiocarbon, dendrochronology, and typological ages for Albert Porter Pueblo, Colorado.

| **Sample** | **Lab #** | **^14^C age yr BP** | **Cal age (2 σ)^a^** | **Probability** |
| --- | --- | --- | --- | --- |
| Bean | Beta 201369 | 760 ± 40 | CE 1203−1294  CE 1191−1199 | 0.990  0.010 |
| Maize | Beta 201368 | 800 ± 60 | CE 1147−1293 CE 1121−1140 CE 1046−1091 | 0.914 0.021 0.065 |
| Maize | Beta 201370 | 880 ± 40 | CE 1234−1243 CE 1037−1225 | 0.016 0.984 |
| Wood | Dendrochronology |  | CE 1110−1260 |  |
| Pueblo III Pottery |  |  | CE 1100−1250 |  |
| Pueblo II Pottery |  |  | CE 920–1140 |  |
| Basketmaker III and Pueblo I Pottery |  |  | CE 600–920 |  |

1. CALIB 7.1 and the IntCal13 Calibration.

Figure 5. Bayesian adjustments of the radiocarbon ages using OXCAL to give the full range of possible ages for Albert Porter Pueblo, Colorado.


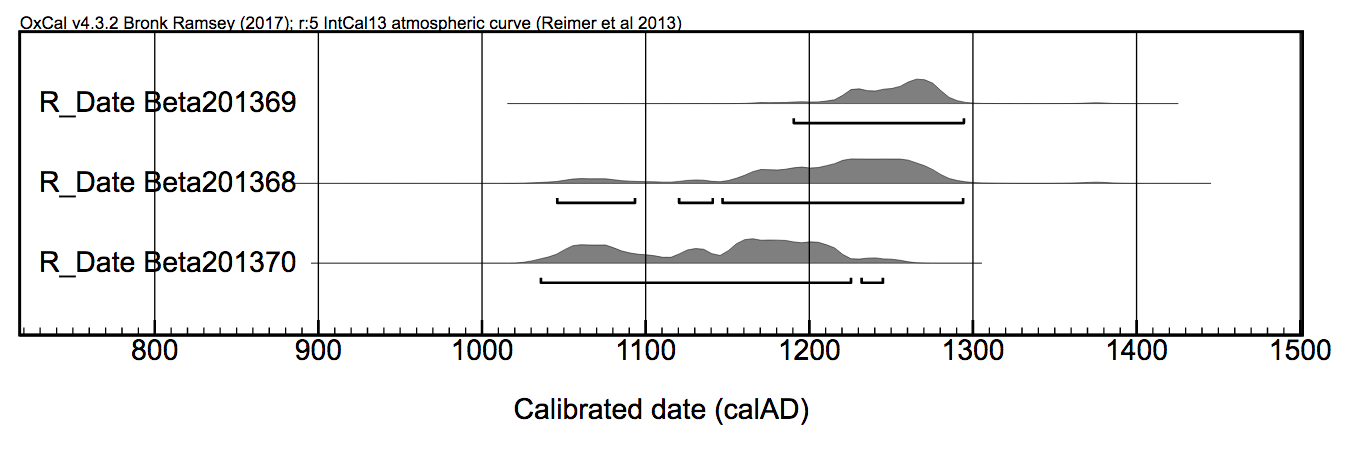


Table 7. Chronostratigraphy and Pt values for Albert Porter Pueblo, Colorado.

| **Depth (m)** | **Texture** | **Munsell Soil Color** | **Age** | **Pt (ppb)^a^** |
| --- | --- | --- | --- | --- |
| 0.0-0.10 | Loam | 7.5YR4/6 |  | 1.8 |
| 0.10-0.18 | Loam | 7.5YR4/6 |  | 1.6 |
| 0.18-0.28 | Silty Clay Loam | 7.5YR5/4 |  | 5.3 |
| 0.28-0.40 | Silty Clay Loam | 5YR5/4 | CE 1191−1294  CE 1046−1293  CE 1037−1243  CE 1110−1260  CE 1100−1250 | 0.7 |
| 0.40-0.50 | Silty Clay Loam | 5R5/4 | CE 920−1140 | 1.4 |
| 0.50-0.55 | Clay Loam | 2.5YR4/6 | CE 600−920 | - |

1. - = below detection.

*Big Bone Lick, Kentucky*

Big Bone Lick is located in the drainage basins of Big Bone and Gum Branch creeks, tributaries to the glaciated Lower Ohio River Valley (Figure 6)^9^. Big Bone Creek and Gum Branch are filled with late Quaternary fluvial sediments including as high-level pre-glacial deposits, two late Pleistocene terraces, and a late Holocene floodplain that is ~6 m thick. The silt-dominated alluvium extends downward from the floodplain surface with abundant wood charcoal, freshwater bivalves, gastropods, and the remains of C_3_ plants, large mammals, and protohistoric to historic contact Fort Ancient, Madisonville Phase pottery (CE 1550−1700) and flaked-stone artifacts (CE 1550−1700)^9^. This stratum represents bison kill and butchering activities, which have been dated to the Little Ice Age (Table 8, Figure 7). They are overlain by a historic stratum contained early 19^th^ century (CE 1810−1850) pottery. Natural stratigraphy was defined on the basis of soil texture and color (Table 9). Sediment samples were collected from intact stratigraphy exposed in an excavation profile wall of a natural floodplain scarp consisting of finely laminated silts and a basal clay. There was no evidence of bioturbation or other natural or cultural disturbances. The stratigraphy of the excavation discussed in this article is illustrated in Figure 6 and is described in detail elsewhere^9^.

Figure 6. Profile of the Big Bone Lick, Kentucky excavation and site location.


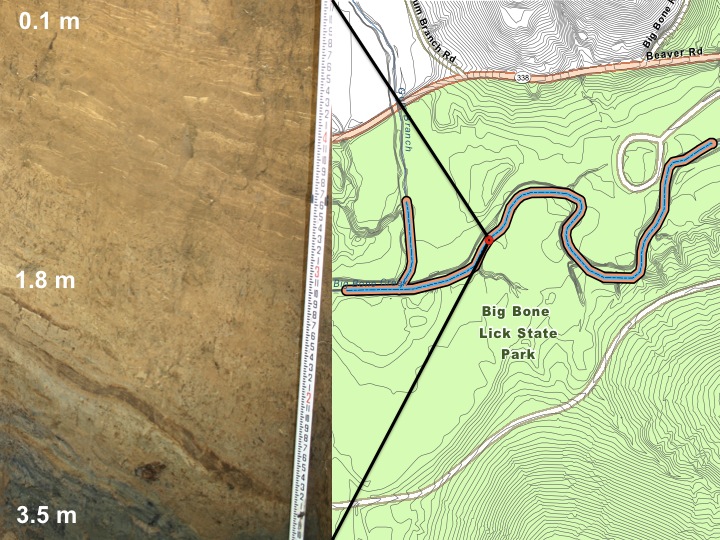


Table 8. Radiocarbon, OSL, and typological ages for Big Bone Lick, Kentucky.

| **Sample** | **Lab #** | **^14^C and OSL age yr BP** | **Cal age (2 σ)^a^** | **Probability** |
| --- | --- | --- | --- | --- |
| Euroamerican Pottery |  |  | CE 1810−1850 |  |
| Collagen (*Bison bison*) | CAMS-161264 | 245 ± 30 | CE 1523−1559  CE 1562−1571  CE 1630−1681  CE 1739−1743  CE 1763−1802  CE 1938−1950 | 0.094  0.007  0.584  0.004  0.267  0.045 |
| Collagen (*Bison bison*) | CAMS-161264 | 260 ± 30 | CE 1519−1593  CE 1619−1670  CE 1779−1799  CE 1943−1950 | 0.297  0.567  0.122  0.014 |
| Collagen (*Bison bison*) | UGa-4291 | 530 ± 105 | CE 1272−1527  CE 1554−1633 | 0.909  0.091 |
| Madisonville Pottery |  |  | CE 1550−1700 |  |
| Fort Ancient Biface |  |  | CE 1550−1700 |  |
| Quartz | UC OSL | 600 ± 200 | CE 1218−1618 |  |

1. CALIB 7.1 and the IntCal13 Calibration.

Figure 7. Bayesian adjustments and the sum of the radiocarbon ages using OXCAL to give the full range of possible ages for Big Bone Lick, Kentucky.


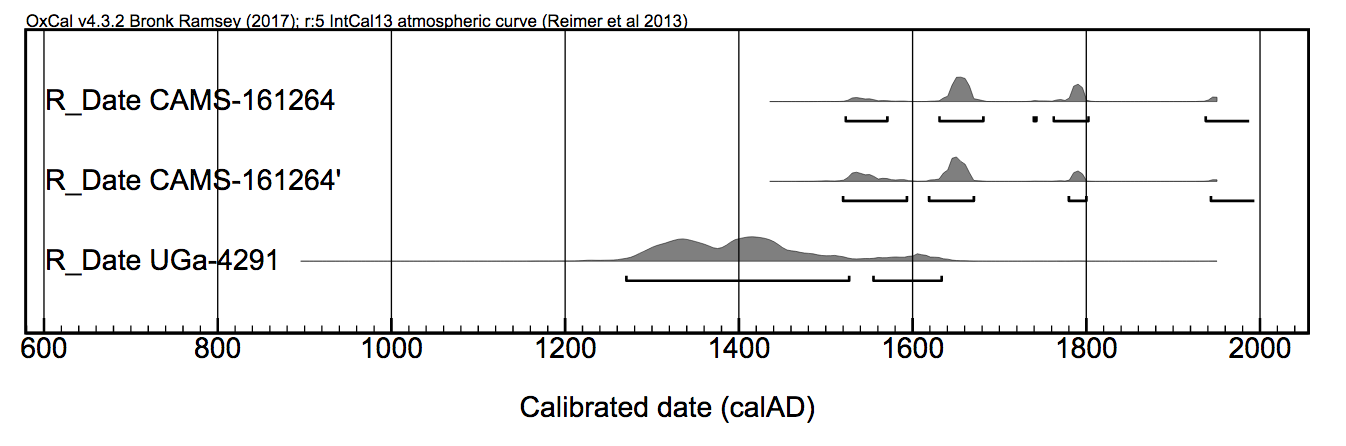


Table 9. Chronostratigraphy and Pt values for Big Bone Lick, Kentucky.

| **Depth (m)** | **Texture** | **Munsell Soil Color** | **Age** | **Pt (ppb)^a^** |
| --- | --- | --- | --- | --- |
| 0.0-1.50 | Friable Silt Clay Loam | 10YR5/4 |  | - |
| 1.50-2.00 | Silt | 10YR5/6 |  | - |
| 2.00-2.25 | Sandy Silt | 10YR6/1 |  | - |
| 2.25-2.50 | Silty Sand | 7.5YR4/2 |  | - |
| 2.50-2.65 | Sand Silt and Clay | 10YR5/3-10YR7/4 | CE 1810−1850 | - |
| 2.65-3.00 | Clay | Gley2 4/5B | CE 1590−1799  CE 1523−1802 | 2.3 |
| 3.00-3.20 | Sand Silt | 10YR4/6 | CE 1515−1700  CE 1550−1700 | - |
| 3.20-3.50 | Clay | Gley2 5/10B | CE 1272−1633  CE 1218−1618 | 4.7 |

1. - = below detection.

*Chaco Canyon, New Mexico*

Chaco Canyon lies within the Chaco Culture National Historical Park, an UNESCO World Heritage site, located within the San Juan drainage basin of northwestern New Mexico (Figure 8). Quaternary deposits in the canyon include two late Pleistocene and early Holocene alluvial terraces and an undifferentiated late Holocene alluvial floodplain, portions of which are covered by colluvium, talus, and aeolian sand^10, 11^. While the canyon has been inhabited since the late Pleistocene, it is best known for the large number of Great Houses constructed by Ancestral Puebloans in a high elevation dry land setting. In addition to these multistory masonry pueblos and kivas, Chaco Canyon includes an extensive network of Ancestral Puebloan canals, dams, furrowed fields, gates, and reservoirs that supplied ample water to maize grown in akchin, dune, and gridded agricultural fields^12^. Sediment samples were obtained from 2 adjacent 1-m^2^ excavations, which cut across an Ancestral Puebloan canal in the Chaco-Escavada Wash confluence area dated to the Pueblo II cultural period (Table 10).^10^ Sediments exposed in the canal excavation document several centuries of use and modification dated using both radiocarbon and optically-stimulated luminescence (OSL) assays; canal excavations and their stratigraphy are described in detail elsewhere.^10^ Little evidence of bioturbation or other types of post-depositional disturbance were evident in the canal strata. Samples were hand collected 10-cm arbitrary levels exposed in and labeled according to their stratigraphic sequence, Munsell soil color, texture (Table 11). The high platinum anomaly (43.6 ppb) at a depth of 50 to 60 cm correlates with the eruptions of the Sunset Crater volcano (Table 11)^11^.

Figure 8. Profile of the Chaco Canyon, New Mexico excavation and site location.


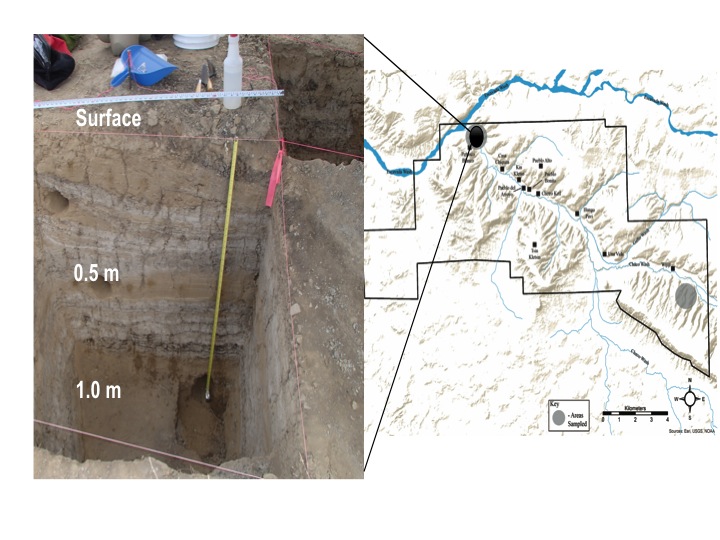


Table 10. Radiocarbon and OSL ages for the Chaco Canyon sediment samples.

| **Sample** | **Lab #** | **^14^C and OSL age yr BP** | **Cal Age (2 σ)^a^** | **Probability** |
| --- | --- | --- | --- | --- |
| Wood Charcoal | UCIAMS 167243 | 985 ± 20 | CE 997−1004 CE 1011−1049 CE 1085−1124 CE 1137−1150 | 0.013 0.665 0.263 0.058 |
| Quartz | UC OSL | 978 ± 60 | CE 976−1096 |  |

1. CALIB 7.1 and the IntCal13 Calibration.

Table 11. Chronostratigraphy and Pt values for Chaco Canyon, New Mexico.

| **Depth (m) Stratum** | **Texture** | **Munsell Soil Color** | **Age** | **Pt (ppb)^a^** |
| --- | --- | --- | --- | --- |
| 0.0-0.10 | Silt Sand Loam | 10YR 5/3 |  | - |
| 0.10-0.20 | Clay Loam | 10YR 4/2 |  | 2.8 |
| 0.20-0.30 | Fine Sand | 10YR 6/4 |  | - |
| 0.30-0.40 | Clay Loam | 10YR 7/2 |  | - |
| 0.40-0.50 | Fine Sandy Silt | 10YR 6/4 |  | - |
| 0.50-0.60 | Clay Loam | 10YR 5/2 | CE 997−1150  CE 976−1096 | 43.6 |

1. - = below detection.

*Serpent Mound, Ohio*

Serpent Mound is a >400 m long serpentine earthwork located on a karst plateau overlooking Brush Creek in southern Ohio (Figure 9)^13, 14^. Caves, sinkholes, and springs are abundant in the immediate vicinity of the earthwork. Meanders in the earthwork form the boundary of sediment-filled sinkholes, which likely held water at the time of construction. While there is some controversy over the exact age of the earthwork’s construction, it overlies an early Woodland cultural component and Fort Ancient radiocarbon ages have been obtained from the earthwork berm^14^. Late Holocene sediment samples were obtained from a 3-m solid sediment core extracted from a 31 x 35 m sinkhole located ~ 100 m south of the earthwork (Table 12). The sinkhole fill represents period of intermittent ponding and drying with inclusions of Early Woodland (800−100 BCE) and Fort Ancient (CE 1000−1650) pot-sherds, flaked-stone artifacts, and abundant wood charcoal. Natural stratigraphy was defined on the basis of soil texture and color (Table 13). The stratigraphy of the core exhibited great integrity with no evidence of bioturbation or other natural or cultural disturbances (Figure 9).

Figure 9. A cross-section of the Serpent Mound, Ohio core and site location highlighting the radiocarbon sample Beta- 467476, CE 896−1021.


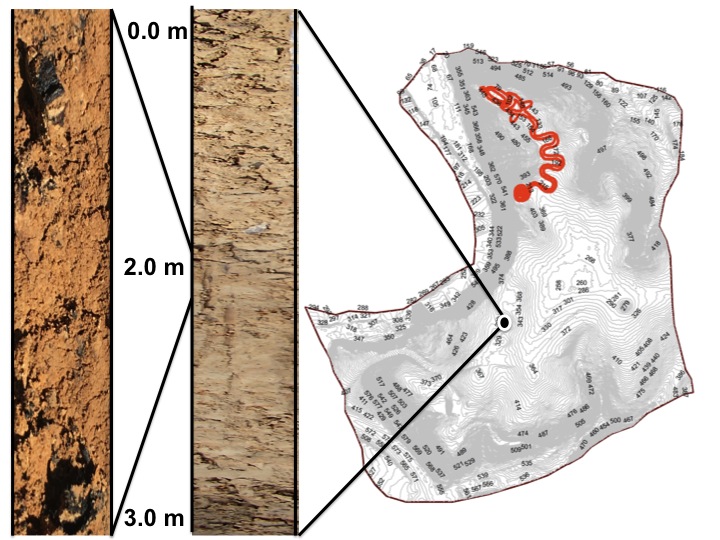


Table 12. Radiocarbon age for the Serpent Mound sinkhole.

| **Sample** | **Lab #** | **^14^C age yr BP** | **Cal age (2 σ)^a^** | **Probability** |
| --- | --- | --- | --- | --- |
| Fort Ancient Pottery |  |  | CE 1000−1650 |  |
| Wood Charcoal | Beta- 467476 | 1070 + 30 | CE 896−927  CE 941−1021 | 0.215  0.785 |
| Early Woodland Pottery |  |  | 800−100 BCE |  |

a. CALIB 7.1 and the IntCal13 Calibration.

Table 13. Chronostratigraphy and Pt values for the Serpent Mound sinkhole.

| **Depth (m)** | **Texture** | **Munsell Soil Color** | **Age** | **Pt (ppb)^a^** |
| --- | --- | --- | --- | --- |
| 0.0-0.10 | Silt Loam | 7.5YR4/4 |  | - |
| 0.10-0.20 | Silt | 7.5YR5/4 |  | 1.8 |
| 0.20-0.25 | Silt Clay | 7.5YR5/4 |  | - |
| 0.25-0.31 | Clay Silt | 7.5YR5/4 |  | - |
| 0.31-0.42 | Clay Silt | 7.5YR5/6 |  | - |
| 0.42-0.50 | Silt Clay | 7.5YR5/6 |  | - |
| 0.50-0.65 | Clay Silt | 7.5YR4/6 |  | - |
| 0.65-0.75 | Silt Clay | 7.5YR4/4 |  | - |
| 0.75-0.92 | Clay Silt | 7.5YR5/4 |  | 0.5 |
| 0.92-1.00 | Silt Clay | 7.5YR4/6 |  | - |
| 1.00-1.25 | Clay | 7.5YR5/6 |  | 1.1 |
| 1.25-1.50 | Silt Clay | 7.5YR6/6 |  | - |
| 1.50-1.62 | Clay Silt | 7.5YR5/6 |  | - |
| 1.62-1.75 | Silt Clay | 7.5YR4/6 |  | 0.9 |
| 1.75-2.00 | Clay | 7.5YR4/6 | CE 1000−1650 | - |
| 2.00-2.25 | Clay | 7.5YR5/6 | CE 896−1021 | 1.2 |
| 2.25-2.45 | Clay | 7.5YR4/4 |  | - |
| 2.45-2.70 | Clay | 7.5YR5/8 |  | - |
| 2.70-2.95 | Stiff Clay | 7.5YR5/8 | 800−100 BCE | - |
| 2.95-3.09 | Stiff Clay | 7.5YR5/6 |  | - |

1. - = below detection.

*Wallace Ruin, Colorado*

Considered a “Chaco Greathouse outlier,” Wallace Ruin is an Ancestral Publoan village located in the McElmo Creek drainage basin, a tributary of the San Juan River in the Mesa Verde region of southwestern Colorado (Figure 10). The site includes Chacoan masonry, large massed buildings, blocked in kivas, an earthen berm and possible reservoir^15^. Construction phases occurred ~ CE 1040, ~ CE 1090, and ~ CE 1120 with a possible abandonment of the village in the middle of the 12^th^ century and reuse during in the 13th century^16^. The age of Wallace ruin was determined on the basis of a detailed ceramic typology (Table 14). The pottery dates to the Pueblo II (~ CE 950–1150) and Pueblo III (~ CE 1150−1350) cultural periods^16^. Sediment samples were obtained from a solid sediment core extracted from the open plaza of the pueblo. Natural stratigraphy was defined on the basis of soil texture and color (Table 15). The stratigraphy of the core exhibited intact stratigraphy with no signs of bioturbation or other natural or cultural disturbances (Figure 10).

Figure 10. A cross-section of the Wallace Ruin, Colorado core and site location.


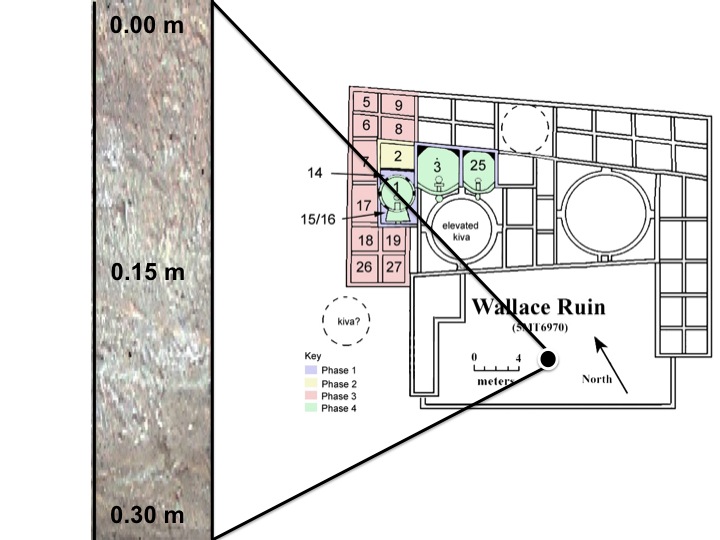


Table 14. Mean Ancestral Puebloan Ceramic ages for the Wallace Ruin, Colorado.^16^

| **Structure** | **# of Samples** | **Stratum** | **Pottery** | **Age** |
| --- | --- | --- | --- | --- |
| 31 | 1 | Surface | Pueblo II | CE 963 |
| 31 | 14 | 1 | Pueblo II | CE 1089 |
| 31 | 7 | 3 | Pueblo II | CE 1105 |
| 31 | 3 | 4 | Pueblo II | CE 983 |
| 31 | 11 | 5 | Pueblo II | CE 1044 |
| 31 | 36 | Combined | Pueblo II | CE 1087 |
| All Units | Mean | Wall Fall | Pueblo III | CE 1165 |
| All Units | Mean | Roof Fall | Pueblo III | CE 1195 |
| All Units | Mean | Cultural Fill | Pueblo III | CE 1168 |
| All Units | Mean | Floor Associations | Pueblo II | CE 1145 |
| All Units | Mean | Subfloor Not Room Associated | Pueblo II | CE 1141 |
| All Units | Mean | Plaza Pre-room Deposits | Pueblo II | CE 1068 |
| All Units | Mean | Wall Fall | Pueblo III | CE 1165 |

Table 15. Chronostratigraphy and Pt values for Wallace Ruin, Colorado.

| **Depth (m)** | **Texture** | **Munsell Soil Color** | **Age** | **Pt (ppb)^1^** |
| --- | --- | --- | --- | --- |
| 0.0-0.10 | Sand Clay Loam | 7.5YR4/4 |  | 2.5 |
| 0.10-0.20 | Sand Clay Loam | 7.5YR3/2 | CE 963−1195 | - |
| 0.20-0.32 | Sand Clay Loam | 7.5YR6/4 | CE 936−1195 | - |
| >0.32 | Coarse-Sand Clay | 5R4/4 | CE 1068 | - |

1. - = below detection.

*Wynema, Ohio, USA*

The Wynema site is a stratified historic contact Fort Ancient village site located on the floodplain of the lower Little Miami River near its confluence with the Ohio River (Figure 11)^17^. The Little Miami floodplain alluvium consists of deep (~ 6 m) finely laminated calcareous silt. The village site is parallel to an abandoned late Holocene channel of the Little Miami River valley. Household features include a longhouse aligned to the summer solstice moonrise, midden deposits with historic contact Fort Ancient, Madisonville Phase (CE 1550−1700) pottery, flaked stone artifacts (CE 1550-1700) and European trade goods (~ CE 1500−1550), abundant late Holocene vertebrates, invertebrates, and carbonized plant remains. These deposits are underlain by a Middle Woodland stratum containing distinctive micro-blade cores (100 BCE−CE 500) at a depth of 1.39-3.42 m. Bison bones, ceramic typology, and an AMS radiocarbon age demonstrate that the site is contemporary with the late Holocene deposits of Big Bone Lick, Kentucky (Table 16). Sediment samples were obtained from natural stratigraphic levels exposed in a 1 x 1 excavation unit. Natural stratigraphy was defined on the basis of soil texture and color (Table 17). While rodent borrows and tree roots were exposed in the excavation, sediment samples were collected from a portion of the profile wall that showed little disturbance from bioturbation or other natural or cultural disturbances.

Figure 11. Profile of the Wynema site, Ohio excavation and site location.


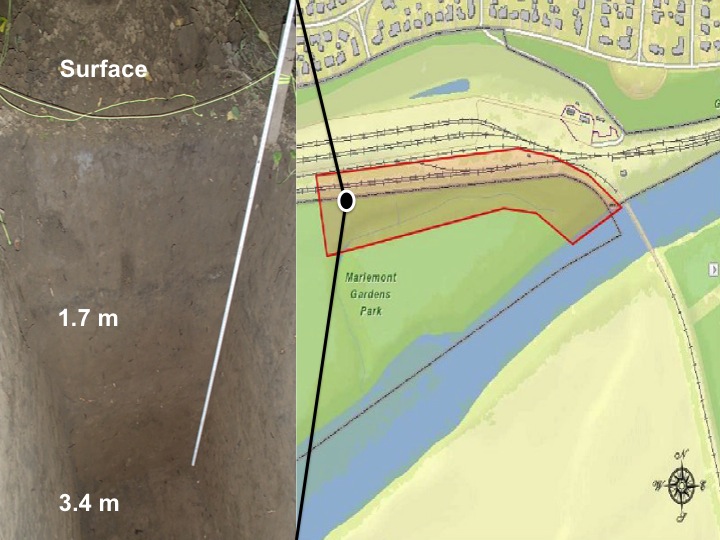


Table 16. Radiocarbon age for the Wynema site, Ohio.

| **Sample** | **Lab #** | **^14^C Age yr BP** | **Cal age (2 σ^a^** | **Probability** |
| --- | --- | --- | --- | --- |
| Madisonville Pottery |  |  | CE 1550−1700 |  |
| Fort Ancient Triangular Biface |  |  | CE 1550-1700 |  |
| Collagen (*Odocoilius* *viginianus*) | Beta-4291 | 370 ± 30 | CE 1447−1527  CE 1553−1633 | 0.575  0.425 |
| Hopewell Micro-blade Core |  |  | 100 BCE−CE 500 |  |

1. - = below detection.

Table 17. Chronostratigraphy and Pt values for the Wynema site, Ohio.

| **Depth (m)** | **Texture** | **Munsell Soil Color** | **Age** | **Pt (ppb)^a^** |
| --- | --- | --- | --- | --- |
| 0.0-0.27 | Silt Clay Loam | 10YR5/3 |  | 2.9 |
| 0.27-0.32 | Mottled Silt Clay Loam | 10YR5/2 | CE 1550−1700  CE 1447−1633 | - |
| 0.32-0.64 | Clay Silt | 10YR4/4 |  | - |
| 0.64-0.87 | Clay Silt | 10YR3/3 |  | - |
| 0.87-1.15 | Sand Clay Silt | 10YR4/3 |  | 0.6 |
| 1.15-1.39 | Sand Clay | 10YR4/3 |  | 1.1 |
| 1.39-3.42 | Clay Silt | 10YR4/3 | 100 BCE−CE 500 | - |

1. - = below detection

**PT Aerosols**

Three types of aerosols significantly affect the Earth's climate, volcanic, desert dust and human-made aerosols created by the burning of coal and oil. The first is the volcanic aerosol layer, which forms in the stratosphere after major volcanic eruptions like Mt. Pinatubo. The dominant aerosol layer is actually formed by sulfur dioxide gas, which is converted to droplets of sulfuric acid in the stratosphere over the course of a week to several months after the eruption^18,19^. Winds in the stratosphere spread the aerosols until they practically cover the globe. Once formed, these aerosols stay in the stratosphere for about two years. They reflect sunlight, reducing the amount of energy reaching the lower atmosphere and the Earth's surface, cooling them. The relative coolness of 1993 is thought to have been a response to the stratospheric aerosol layer that was produced by the Mt. Pinatubo eruption. In 1995, though several years had passed since the Mt. Pinatubo eruption, remnants of the layer remained in the atmosphere. Data from satellites such as the NASA Langley Stratospheric Aerosol and Gas Experiment II have enabled scientists to better understand the effects of volcanic aerosols on our atmosphere^20^.

The aerosol at Erebus volcano in Antarctica is also distinct in that the gas emissions have some of the highest measured halogen/SO_2_ ratios in the world as well as an abundance of heavy metals^21^. Furthermore, most of the aerosol mass appears to be narrowly confined to particles with diameter of ∼0.1 mm, substantially finer than often observed at other volcanoes, which indicates it can be widely distributed by prevailing wind patterns.

Studies of volcanic aerosols from Vulcan Masaya, Nicaragua, for example, also indicate that platinum group element (PGE) concentrations (Re-Os-Ir-Rh-Ru-Pt-Pd) are significantly higher than in urban air^22^. If PGE concentrations measured in volcanic aerosols from Vulcan Masaya are typical of volcanic aerosols in general, volatile PGE emissions are globally significant and merit detailed study. The magmatic fractionation of Re-Os-Ir-Rh-Ru-Pt-Pd is governed by the volatility of relevant PGE-containing complexes (e.g., oxides, hydrogen halides, sulfides) and the physicochemical properties of the magma (temperature, fugacities of relevant chemical species). Preliminary data for volcanic aerosols from Vulcan Masaya, Nicaragua, indicate that the PGE abundance patterns are strongly and uniquely fractionated (i.e., high Pd/Pt, Os/Ir, Ru/Rh) compared to other important sources of PGE. These patterns may serve as a unique fingerprint for volcanic sources of PGEs, provided they are typical for volcanic exhalations in general. If PGE abundance patterns are a unique geochemical indicator of PGE sources in the geologic record, they may have potential applications ranging from the identification of disputed impact layers to assessing origins of PGE concentrations in the environment.

**Tephra Volume**

Volume determination of tephra deposits is necessary for the assessment of the dynamics and hazards of explosive volcanoes. One of the main ways volcanologists categorize the volume and explosivity of the world’s major volcanic eruptions is through the analysis of tephra distribution. This is because tephra deposits retain a large amount of important information related to the dynamics and physical parameters of the associated volcanic eruptions. One of the most important parameters that can be derived from the analysis of tephra deposits is the erupted volume, which is essential for the assessment of the associated hazards Nonetheless, the calculation of erupted volume is complicated by (1) the nonuniversal relationship of the deposit thinning with distance from the vent, (2) the poor preservation and accessibility to significant parts of tephra deposits (limited outcrops and/or tephra dispersal often over large water bodies), and (3) the difficulty in extrapolating thickness decay patterns of the medial portion of deposit, which is typically well preserved, to both proximal and distal areas. For example, the consequences of a future, caldera-forming eruption from the Yellowstone volcano have been the subject of much speculation but little quantitative research in terms of regional ashfall impacts.

Despite graphic and often fanciful media depictions of the devastation and the impact on human life that would result from a modern supereruption (producing >1000 km^3^ volcanic ash or >400 km^3^ dense rock equivalent of magma), no historical examples exist from which to draw comparison. The largest eruptions of the past few centuries have produced a few to several tens of cubic kilometers of magma. Examples include Tambora volcano, Indonesia in 1815, Krakatau in 1883, the Katmai/Valley of Ten Thousand Smokes eruption, Alaska in 1912, Quizapu volcano, Chile in 1932, and most recently, Pinatubo, Philippines in 1991. These erupted volumes are much larger than the Mount St. Helens eruption in 1980 (0.2–0.4 km^3^), but at least an order of magnitude smaller than the largest Yellowstone events^23^.

Several empirical volume calculation methods have been proposed over the past 40 years, ranging from the analysis of crystal/glass ratio of large pumices to various integration methods of thickness-versus distance- from-the-vent relations. For example, in the method described by), a sensitivity analysis was carried out on two deposits of different magnitude^24^. The determination of tephra-deposit volumes is crucial to the characterization of active volcanoes, with obvious implications for environmental and climatic impact, estimation of magma production rate, long-term hazard assessments, and forecasting of future eruptions.

For volcanic ash in the atmosphere, it is difficult to use a universal detection because the ash particle radius in the cloud usually varies from 1 to 15 μm and the chemical properties may vary from one volcano to another. The ash cloud is tracked using the brightness temperature difference for several days, but beyond that the detection is generally not reliable. Computed model results of the vertical distribution of SO_2_, volcanic ash mass and particle number concentration give further insight into the atmospheric dispersion and removal processes after the eruption of Kasatochi volcano. It should be noted that the brightness temperature difference signal does not correlate with the ash content, however, comparing modeled ash column concentration with modeled data plots shows qualitative agreement in the travelling routes. Comparisons show fairly clearly that the ash cloud travelled along the same pathways as SO_2_^25^. Therefore, the assumption of the same initial percentage vertical distribution for volcanic ash and SO_2_ is justified for the model simulations.

**References**

1. Siegel, P. E., J. G. Jones, D. M. Pearsall, N. P. Dunning, P. Farrell, N. A. Duncan, J. H.

Curtis, and S. K. Singh. Paleoenvironmental Evidence for First Human Colonization of the Eastern Caribbean. *Quaternary Science Reviews* 129: 275–295 (2015).

2. Jones, J. G., N. P. Dunning., N. Duncan, D. Pearsall, and P. Siegel. “Antigua.” In: Siegel,

P. (Ed.). *Island Historical Ecology: Socionatural Landscapes of the Eastern and Southern Caribbean*, Pp. 239-269. New York: Berghahn (2018).

3. Wells, E. C., S. M. Pratt, G. L. Fox, P. E. Siegel, N. P. Dunning, and A. R.

Murphy. Plantation Soilscapes: Initial and Cumulative Impacts of Colonial Agriculture in Antigua, West Indies. *Environmental Archaeology* 22:23-35 (2018).

4. Dunning, N. P., R. Griffin, J. Jones, R. Terry, Z. Larsen, and C. Carr. Life on the

Edge: Tikal in a Bajo Landscape. Lentz, D., N. Dunning, and V. Scarborough (eds). *Tikal: Paleoecology of an Ancient Maya City*, Pp. 95-123, Cambridge: Cambridge University Press (2015).

5. Scarborough, V., N. P. Dunning, K. Tankersley, C. Carr, E. Weaver, L. Grazioso,

B. Lane, J. Jones, P. Buttles, F. Valdez, and D. Lentz. Water and Sustainable Land Use at the Ancient Tropical City of Tikal, Guatemala. *Proceedings of the National Academy of Sciences*: 12408-12413. (2012).

6. Culbert, T. P., Análisis de Cerámica. In: Proyecto de Silvacultura y Manejo de Aguas de los Antiguos Mayas de Tikal: Temporada de 2009, Lentz, D.L., Grazioso Sierra, L., Dunning, N.P., Scarborough, V.L., *Proyecto de Silvacultura y Manejo de Aguas de los Antiguos Mayas de Tikal: Temporada de 2009,* Pp. 67-75. Dirección Patrimonio Cultural y Natural de Guatemala. (2009).

7. Scarborough, V. L., L. Grazioso Sierra, N. P. Dunning, B. Lane, and E. Weaver.

Manejo de Aguas en Tikal. Lentz, D.L., Grazioso Sierra, L., Dunning, N.P., Scarborough, V.L., *Proyecto de Silvacultura y Manejo de Aguas de los Antiguos Mayas de Tikal: Temporada de 2009,* Pp. 30-75. Dirección Patrimonio Cultural y Natural de Guatemala. (2009).

8. Ryan, S.C. *The Archaeology of Albert Porter Pueblo (Site 5MT123): Excavations at a*

*Great House Community Center in Southwestern Colorado.* Crow Canyon Archaeological Center, Cortez (2015).

9. Tankersley, K.B., Murari, M.K., Crowley, B.E., Owen, L.A., Storrs, G.W., Mortensen, L.

Quaternary chronostratigraphy and stable isotope paleoecology of Big Bone Lick, Kentucky, USA, *Quaternary Research* **83**, 479–487 (2015).

10. Scarborough, V.L, S. Fladd, N.P. Dunning, S. Plog, L.A. Owen, C. Carr, K.B.

Tankersley, J.P McCool, A. Watson, E. Haussner, B. Crowley, K. Bishop, D. Lentz, and R. G. Vivien. Water uncertainty and ritual predictability at Chaco Canyon, New Mexico. *Antiquity*. (2018) In press.

11. Tankersley, K.B., Huff, W.D., Dunning, N.P., Owen, L.A., Scarborough, V.L. Volcanic

minerals in Chaco Canyon, New Mexico and their archaeological significance. *Journal of Archaeological Science: Reports* **17**, 404-421 (2018).

12. Vivian, R.G. *The Chacoan Prehistory of the San Juan Basin*. Academic Press, New

York (1990).

13. Dalby, T.S. *Geological Aspects of Key Archaeological Sites in Northern Kentucky and*

*Southern Ohio*, Ohio Geological Survey, Columbus (2007).

14. Lepper, B., Duncan, J., Diaz-Granádos, C., & Frolking, T. Arguments for the Age of

Serpent Mound. *Cambridge Archaeological Journal,* 1-18. doi:10.1017/S095977431800001X (2018).

15. Varien, M.D., Lipe, W.D., Adler, M.A., Thompson, I.M., Bradley, B.A., Southwestern

Colorado and Southeastern Utah Settlement Patterns: A.D. 1100 to 1300, Adler, M.A., (Ed.) *The Prehistoric Pueblo World, A.D. 1150-1350*, Pp. 86-113, University of Arizona Press, Tucson.

16. Bradley, B.A. *Excavations at Wallace Ruin (5MT6970) Montezuma*

*County, Colorado 1998-2010*. University of Exeter, Exeter (2010).

17. Tankersley, K. B. and R. Newman *Dr. Charles Louis Metz and the American Indian*

*Archaeology of the Little Miami River Valley.* Little Miami Publishing Company, Milford (2016).

18. Giammanco S, Sims, K.W.W. Neri, M..  Measurements of ^220^Rn and ^222^Rn

and CO_2_ emissions in soil and fumarole gases on Mt. Etna volcano (Italy): Implications for gas transport and shallow ground fracture. *Geochemistry, Geophysics, Geosystems*, **8**, 10:1644 (2007).

19. Villemant, B., Salaün, A., Staudacher, T., Evidence for a homogeneous

primary magma at Piton de la Fournaise (La Réunion): A geochemical study of matrix glass, melt inclusions and Pélé's hairs of the 1998–2008 eruptive activity. *Journal of Volcanology and Geothermal Research* **184**, 79–92 (2009).

20. Moune, S., Faure, F., Gauthier, P.J., Sims, K.W.W., "Pele’s hairs and tears:

natural probe of volcanic plume," *Journal of Volcanology and Geothermal Research*, **164**, 244-253 (2007).

21. Ilyinskaya, E., Oppenheimer, C., Mather, T.A., Martin, R.S., Kyle, P.R., Size‐

resolved chemical composition of aerosol emitted by Erebus volcano, Antarctica. *Geochemistry, Geophysics, Geosystems*, **11**, (3) 1-14 (2010).

22. Mills, M. J., Schmidt, A., Easter, R., Solomon, S., Kinnison, D.E., Ghan, S.J., Neely,

R.R., Marsh, D.R., Conley, A., Bardeen, C.G., Gettelman, A., Global volcanic aerosol properties derived from emissions, 1990–2014, using CESM1(WACCM), *Journal of Geophysical Research: Atmosphere*, **121**, 2332–2348 (2016).

23. Bonadonna, C., and Houghton, B.F., Total grain-size distribution and volume of

tephrafall deposits. *Bulletin of Volcanology*, **67**, 441-456 (2005).

24. Christiansen, R.L and Blank, H.R. Jr., *Volcanic stratigraphy of the Quaternary*

*rhyolite plateau in Yellowstone National Park, Wyoming*: U.S. Geol. Survey Prof. Pap. 729-B, 18 (1972).

25. Stohl, A., Prata, A.J., Eckhardt, S., Clarisse, L., Durant, A., Henne, S., Kristiansen, N.I.,

Minikin, A., Schumann, U., Seibert, P., Stebel, K., Thomas, H.E., Thorsteinsson, T., Tørseth, K., Weinzier, B., Determination of time- and height-resolved volcanic ash emissions and their use for quantitative ash dispersion modeling: the 2010 Eyjafjallajökull eruption, Atmospheric Chemistry and Physics, **11**, 4333–4351 (2011).
